# A Software Architecture for Adaptive Modular Sensing Systems

**DOI:** 10.3390/s100807514

**Published:** 2010-08-10

**Authors:** Andrew C. Lyle, Michael D. Naish

**Affiliations:** Sensing and Mechatronic Systems Laboratory, Department of Mechanical and Materials Engineering, The University of Western Ontario, London, Ontario, N6A 5B9, Canada; E-Mail: andrew.lyle@gmail.com

**Keywords:** adaptive sensing systems, intelligent sensors, modular sensors, sensor reconfiguration, template algorithms, software architecture, knowledge representation, active sensors, transducer interface

## Abstract

By combining a number of simple transducer modules, an arbitrarily complex sensing system may be produced to accommodate a wide range of applications. This work outlines a novel software architecture and knowledge representation scheme that has been developed to support this type of flexible and reconfigurable modular sensing system. Template algorithms are used to embed intelligence within each module. As modules are added or removed, the composite sensor is able to automatically determine its overall geometry and assume an appropriate collective identity. A virtual machine-based middleware layer runs on top of a real-time operating system with a pre-emptive kernel, enabling platform-independent template algorithms to be written once and run on any module, irrespective of its underlying hardware architecture. Applications that may benefit from easily reconfigurable modular sensing systems include flexible inspection, mobile robotics, surveillance, and space exploration.

## 1. Introduction

### 1.1. Sensors and Actuators in Industry

Sensors and actuators have seen widespread utilization in many of today’s industrial processes. These devices convert physical phenomena to and from electrical signals for the purpose of measurement, tracking, and/or control by way of digital devices such as microcontrollers, programmable logic controllers (PLCs), and mainstream computers. In current practice, fixed combinations of sensors and actuators are typically employed, with each combination often deployed in a static orientation and tailored to fulfil a specific application.

In order to enhance accuracy and reliability in such applications, multiple sensors are often combined into composite entities. For example, unlike a single camera, two or more cameras operating in tandem could effectively form a sensor capable of depth perception through sensor fusion. Sensors that detect different, but related, types of physical phenomena may also be combined to produce a new device that produces measurements that are more accurate than either of its constituent sensors are capable of providing. For example, a thermocouple could be combined with an infrared camera to increase the accuracy of sensed temperature.

The sizes of the transistors used in the implementation of microprocessors and other integrated circuits through *very large-scale integration* (VLSI) are becoming ever smaller, consistent with *Moore’s Law* [1], due to advancements in semiconductor fabrication techniques. The sizes of sensors and actuators are also being reduced at an equally rapid rate due to advancements in *microelectromechanical systems* (MEMS) and *nanoelectromechanical systems* (NEMS) fabrication techniques. As a result of these technological advancements, it has become quite practical to combine sensing, actuation, processing logic, as well as transceivers that provide wired and wireless networking capability into a single monolithic device termed a *smart transducer*. With the ability to transmit information and locally execute algorithms independently, without depending upon a larger, static, and more powerful mainstream computer system, the potential for smart transducers to collaborate amongst themselves without any external influence in order to achieve a specific goal becomes worthy of consideration.

### 1.2. The Need to Combine Sensors and Actuators

Sensing systems designed to be operated in a static orientation and under controlled operating conditions cannot be cheaply or quickly reconfigured to handle a change in process requirements, such as in assembly lines where the product being assembled changes completely or is now required to be processed in previously unconsidered orientations. Instead of merely considering each existing sensor as a strictly self-contained device that is to be utilized in an exclusive scenario, or in tandem with others sensors, each sensor may be enhanced through physical combination with one or more actuators in addition to other sensors, resulting in an *active* sensing device.

Combining a sensor with an actuator greatly enhances the ability of the sensor, which is now augmented with mobility and gains the ability to adapt to changing process requirements, such as monitoring non-stationary objects of interest. For example, a camera could be mounted on a rotational stage to form a panoramic camera with a field of view of 360 degrees, enabling it to track objects that move anywhere within a particular plane. The relocation of processing logic directly onto the hardware comprising a smart transducer allows such a composite sensing device to be completely self-contained, and scalable to even larger combinations of modules.

The ability to combine diverse modular sensor and actuator components to produce flexible modular sensor systems facilitates rapid reconfiguration to suit any requirement, and is a technique that will prove useful in many modern applications. Examples of applicable domains include flexible inspection, mobile robotics, surveillance, and even space exploration.

### 1.3. Research Objective

The aim of this work is to develop a software architecture and knowledge representation scheme that facilitates the flexible, scalable, and reliable combination of modular sensing and actuation components for the purpose of forming composite sensing devices with motion capability. Each modular component provides a core sensing or actuation functionality (such as temperature or pressure measurement) and contains embedded knowledge of its capabilities (such as its operating range and response time), which is communicated to other modules within its environment. The design of the architectural framework should fulfil the following criteria:

- **Heterogeneity**—Support the connection of sensor and actuator modules possessing diverse functionality and capabilities.
- **Autonomy**—Support the autonomous discovery of the capabilities of networked modules, and the autonomous configuration of these modules based on their discovered capabilities.
- **Pose/Geometry Determination**—Support the determination of the absolute or relative *pose* (position and orientation) of individual modules, and by extension the overall geometry of a set of connected modules.
- **Assumption of a Collective Identity**—Facilitate the assumption of a collective identity by successfully connected modules, based on their capabilities and relative positions and orientations.
- **Process Distribution**—Support the splitting and distribution of a complex task among a group of networked modules.
- **Resource Management**—Manage the hardware resources on each module in an efficient, intuitive, and simple manner.
- **Scalability**—Maintain reliable operation with an increasing number of connected sensor and actuator modules.
- **Robustness**—Adapt automatically to the addition, removal, or failure of modules in real-time.

## 2. Survey of Related Work

### 2.1. Logical Sensor Architectures

Modular sensing systems are often composed of a number of sensors and possibly actuators of diverse types. Enabling intercommunication among these transducers, especially in a manner facilitating easy reconfigurability, is often problematic due to the various analog and digital interfaces through which communication must take place. Therefore, facilitating interoperability between the devices often requires interface-specific solutions that may become unwieldy when reconfiguration of large sensor-actuator systems is required.

**Figure 1.**
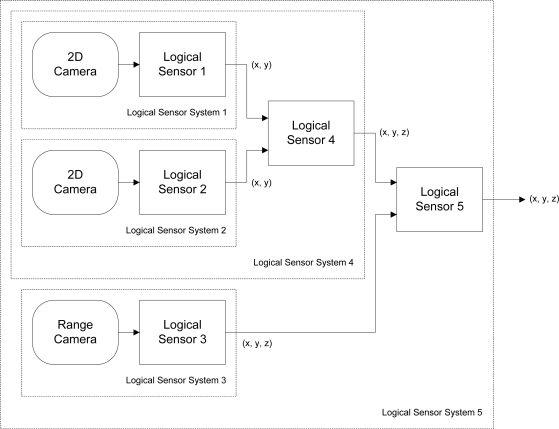
Sample logical sensor hierarchy [3].

One approach that aims to simplify the assembly of multi-sensor systems, aspects of which are utilized in the design of the software architecture described in this paper, is the *Logical Sensor Specification* (LSS) [2,3]. The LSS introduces the useful abstraction of a *logical sensor*, which packages the data produced by diverse sensor types into a uniform digital representation. Thus, the internal hardware implementation of a sensor and its interface are completely encapsulated, facilitating easy assembly of dynamically reconfigurable modular sensing systems. A logical sensor need not necessarily be associated with a physical entity, and may even simply be software that satisfies the abstraction interface. Hierarchies of logical sensors may even be assembled to form a composite logical sensor that operates as a single entity. An example of such a hierarchy is shown in Figure 1, in which a logical three-dimensional measurement sensor is implemented.

Another existing architecture that provides similar benefits to that of a logical sensor hierarchy is that of *logical neighbourhoods* [4,5]. In this architecture, sensor and actuator nodes are abstracted into uniform *virtual nodes* that may in turn be further abstracted into a composite collection termed a *logical neighbourhood*. Logical neighbourhoods appear as a single virtual node entity that may be further composed into larger neighbourhoods. Virtual nodes higher in the hierarchy transmit commands and data to nodes lower in the hierarchy through a wireless interface. Virtual nodes and logical neighbourhoods are specified through templates written in the *SPIDEY* declarative language [4,5].

### 2.2. The IEEE 1451 Standards

The knowledge representation scheme utilized with the software architecture to represent the functionality and capabilities of modular sensing and actuation components utilizes aspects of the NIST *IEEE 1451* [6,7] family of standards for smart transducers. These standards describe a set of network-independent communication interfaces that simplify the connection of sensors or actuators to microprocessors, instrumentation systems, and networks, enabling them to be utilized in a “plug-and-play” manner. The core feature of these standards is the *Transducer Electronic Data Sheets* (TEDS) defined for each transducer type, which is a local or remote region of memory that stores information about the functionality and capabilities of the transducers in an easily accessible and network-independent form.

### 2.3. Existing Modular Sensing Systems

A number of implementations of reconfigurable modular sensing systems exist in which smart sensor and actuator components may be combined or otherwise collaborate. A popular implementation of a reconfigurable modular sensing system is the UC Berkeley *Mica* platform [8]. Each Mica node, known as a *mote*, measures 1.25 × 2.25 inches and runs the TinyOS real-time operating system [9]. Although the motes are capable of collaboration through the use of a peer-to-peer multi-hop wireless networking protocol, no actuation capabilities are supported, and therefore the motes are limited to operating in non-active sensing applications. A similar project is the *Smart-Its* [10] project. Smart-Its are self-contained nodes, as small as 17 × 25 × 15 mm, designed to be stuck onto everyday objects. The objects are thus enhanced with sensing and computational capabilities. Each Smart-Its node is aware of its attached sensors and is capable of wirelessly relaying this information as well as sensed data to other nodes in its environment on demand. The nodes are able to process data locally, as well as relay information to higher-end computing devices. However, like the Mica motes, the active operation and automatic reprogramming capability of Smart-Its nodes is limited.

More closely related to the system described in this paper are the *eBlocks* [11,12] embedded system building blocks. An eBlock is an electronic module, incorporating a PIC microcontroller, that facilitates the construction of simple sensor networks even by users who are not technically adept. Unlike general purpose nodes such as the Mica motes, and like the system described in this paper, each eBlock performs a specific, well-defined function. These functions include sensing, actuation, boolean logic, and wireless communication. Although reconfigurable, connected blocks are unable to determine their overall geometry or automatically assume a collective identity to suit new configuration requirements. The possible applications of the system are also limited due to the usage of simple combinational and sequential logic functions to produce composite readings and actions. Similarly, the *I-BLOCKS* project [13], LEGO DUPLO bricks are populated with a PIC16F876 microcontroller as well as select sensors and actuators. When physically connected, the blocks are able to communicate through a physical half-duplex connection or wirelessly. The blocks are also capable of some positional awareness through sensor fusion of the readings produced by multiple infrared sensors. However, like the eBlocks, connected blocks are unable to determine their overall geometry or automatically assume a collective identity based on their orientations. The blocks are also not designed to be easily reprogrammed to suit changing application requirements.

*MASS* (Modular Architecture for Sensing Systems) [14] is a modular sensing system architecture optimized for low power consumption and is based on hot-pluggable intelligent nodes. Four types of MASS modules are available which provide powerful processing capability, sensing capability with rudimentary local data analysis, wireless inter-node communication, or power. Upon connection, modules within a particular node detect each other’s resources and the node assumes an appropriate behaviour profile. Similar to the software architecture described in this paper, the MASS software architecture is a layered architecture based on the *Open Systems Interconnection* (OSI) reference model [15], and contains a message-based API (Application Programming Interface) for inter-node communication. Exchange of IEEE 1451-compliant datasheets is also supported. However, MASS provides no capability for active nodes nor the assumption of behaviour profiles based on node positions and orientations.

*BUG* [16] is a powerful ARM11-based modular sensing system platform consisting of a collection of modules that are designed to be snapped together. Among the available modules are LCD, motion detection, GPS, and camera modules. Although extremely flexible, the BUG platform does not currently provide functionality to facilitate active sensing. In addition, the orientations in which BUG modules may be connected are still somewhat limited, and cannot be determined, thus restricting the ability of a composite BUG system to form a new collective identity based on the orientation of its constituent components.

*Posey* [17] is a hub-and-strut construction kit enhanced with computational ability. Within a Posey assembly, hubs and struts are optocoupled into flexible ball-and-socket joints. An ATmega168 microcontroller in each hub and strut captures data from the optocoupled connections, and uses it to determine the geometric configuration of the joint. This information is then relayed wirelessly to a remote personal computer for further processing. Although Posey supports the acquisition of position and orientation information from the local processing unit located within each ball-and-socket joint, the units themselves do not locally collaborate to form a composite entity. Rather, the system depends upon a more powerful mainstream computer system to provide the necessary intelligence to compose the data provided from the joints.

## 3. Architecture Description

### 3.1. Module Hardware Overview

The basic module used to construct modular sensing systems is the *transducer interface module* (TIM). Each is capable of a single sensing or actuation function, and is uniquely identified by a 64-bit address. As specified in the IEEE 1451 standard for smart transducers [6], each module possesses one or more *Transducer Electronic Data Sheet* (TEDS) specifications in non-volatile memory, from which a description of the characteristics of its associated sensors or actuators may be obtained.

**Figure 2.**
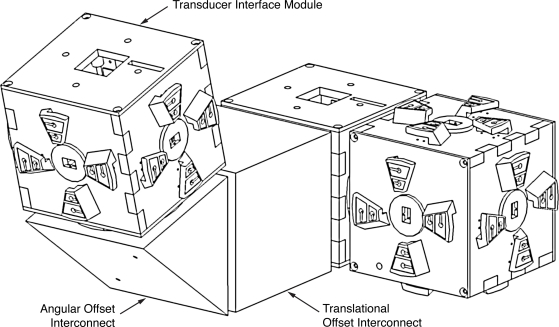
Transducer Interface Modules and interconnects.

**Figure 3.**
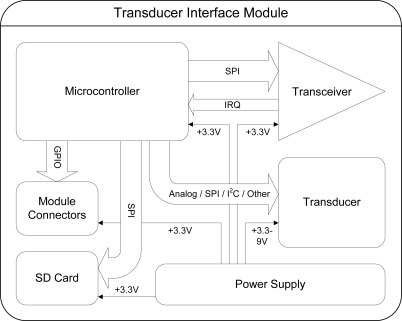
Transducer Interface Module block diagram.

TIMs are cubical in shape, and thus each possesses six faces to which up to five other modules may be connected, as shown in Figure 2. One face is reserved for use by the transducer associated with the module. The hardware which comprises a TIM, shown in Figure 3, includes the associated transducer; a high-speed NXP Semiconductors LPC2148 ARM-based microcontroller [18]; a Nordic Semiconductor nRF24L01 [19] wireless transceiver supporting high-speed data transmission, multi-channel operation and carrier detection; a Secure Digital^™^ (SD) flash memory card providing high-capacity, non-volatile storage for data and algorithms; a power supply capable of providing a voltage of 3.3 volts to 9 volts; and five *module connectors*, which are proprietary interfaces used to physically connect additional modules. The interfaces are designed such that the relative orientation between any two connected modules may be determined. Further details on the electrical and mechanical design aspects of the TIMs may be found in [20].

### 3.2. Other Module Types

A modular sensing system may consist of two other types of modules significant to the software architecture. These modules perform tasks unrelated to sensing and actuation; instead, they support the inter-operation of a group of TIMs.

#### 3.2.1. Administration Module

An *administration module* is used by the system user to detect and manage TIMs within its vicinity. It possesses only a power supply, a microcontroller, and a transceiver. It may be integrated into a complete computer system, or be a small, self-contained console with a user interface. Administration modules may also act as a sink for transducer readings and as a gateway for communication with a larger network, such as the Internet.

#### 3.2.2. Interconnect Module

*Interconnect modules* are each built to assume one of a variety of non-standard shapes, and are used to provide angular and translational offsets between connected TIMs which would otherwise not be possible due to the cubical shape of the TIMs. An example of an interconnect module which provides an angular offset is shown in Figure 2. They possess only a microcontroller and module connectors, and draw power from the TIMs to which they are connected. The nature of the offset provided by a particular interconnect module is stored in its TEDS, and may be accessed through its module connectors.

### 3.3. Software Architecture Stack

**Figure 4.**
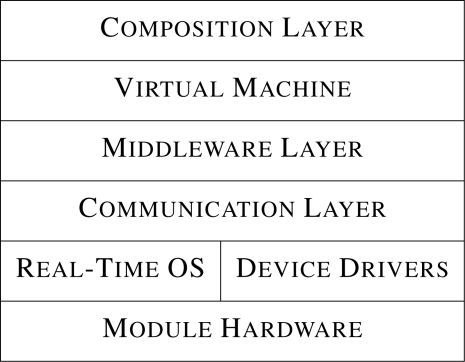
Software architecture stack.

The software architecture described in this paper is a distributed architecture based on the *Open Systems Interconnection* (OSI) reference model [15], and consists of six layers (one of which is divided into two sub-layers) as shown in Figure 4. The use of a *distributed* architecture ensures that no single point of failure exists within a modular sensing system and also facilitates architecture scalability, unlike *centralized* architectures, in which a single point of failure is often introduced that can also limit scalability in large systems where communication between nodes mostly occurs through this point.

The use of a layered architecture model allows the implementation of any layer to change independently of the others, since the implementation of each layer is encapsulated from the layer above, to which it provides service. This information-hiding technique also facilitates a more robust software architecture, and makes each of the architecture layers easier to implement, modify, and debug. The function of each layer is defined as follows:

#### 3.3.1. Module Hardware

Contains the physical components of a module needed for execution of the operating system, sensing and actuation functionality, as well as wired and wireless communication.

#### 3.3.2. Real-Time Operating System/Device Drivers

Provides resource management functionality and an environment for concurrent task execution.

#### 3.3.3. Communication Layer

Provides an interface to the wireless transceiver driver that automatically accounts for transmission problems such as packet loss and synchronization. This layer also provides an interface through which modules may communicate using their face connectors.

#### 3.3.4. Middleware Layer

Provides the commands and services through which the member TIMs comprising a *logical module* may interact and communicate with each other in order to achieve a specific goal. A logical module is an abstraction of one or more collaborating TIMs.

#### 3.3.5. Virtual Machine

Provides a platform-independent execution environment for the algorithms utilized in the composition layer. Platform independence is facilitated through the use of a compact implementation of Sun Microsystems’ *Java Virtual Machine* [21].

#### 3.3.6. Composition Layer

Encompasses one or more *logical module template classes* that provide the intelligence necessary for a group of collaborating TIMs to behave as a logical entity. Each template algorithm is accompanied by a *logical module template TEDS* that describes the basic characteristics of a logical module entity derived from on it.

#### 3.4. Real-Time Operating System

The software architecture utilizes a *real-time operating system* (RTOS), which enables it to be implemented in a modular fashion through the concurrent execution of various *tasks*. As a result, the management of the hardware resources of a module, as well as the development and debugging of the software architecture, is vastly simplified. Tasks are implemented as independent functions that appear to be running simultaneously, but are actually sharing the execution time of the microcontroller through the use of *scheduling* mechanisms implemented within the operating system.

In an RTOS, concurrently executing tasks may be scheduled using either a *pre-emptive* scheduling policy or a *cooperative* scheduling policy. In pre-emptive scheduling, CPU time is automatically shared between tasks based on their assigned priority, while in cooperative scheduling each task maintains control of the CPU until it explicitly yields control. Pre-emptive scheduling is advantageous since it prevents long-running, low-priority *background* tasks from blocking shorter, higher-priority *foreground* tasks from executing, thus improving system response speed to external events. In the popular *TinyOS* [9] RTOS, which utilizes a cooperative scheduler, all tasks must run to completion. Long-running background tasks are therefore prohibited, and care must be taken to ensure that each task completes in a reasonable amount of time.

Standard background tasks executed upon startup and initialization of a TIM are the *network communication task*, which performs various duties related to communication on the various wireless data channels; the *face communication task*, which manages the communication of the TIM with others physically connected to its faces and calculates their relative *pose* (position and orientation); the *administrative interface task*, which allows the system user to monitor and administrate any physical module, or logical group of modules, within the modular sensing system; and at least one *message handler task*, which process messages received by a TIM related to its local hardware or a logical module of which it is a member.

The real-time operating system chosen for use in the software architecture presented herein is *TNKernel* [22]. This RTOS was chosen because it is free, open source, compact, well documented, and contains a priority-based pre-emptive task scheduler. TNKernel also makes provisions for message passing and synchronization between concurrently executing tasks.

### 3.5. File System

A *file system* is a set of data structures that facilitates the storage, organization, and retrieval of files from a data storage device. A file system is employed within the software architecture to provide an efficient, high-level interface to information and algorithms stored on SD flash cards that determine the identity and behaviour of a particular module in a network. These SD flash cards are formatted with the *FAT32* (32-bit File Allocation Table) [23] file system and initialized with a standard file structure. The FAT32 file system was chosen since it is widely supported, stable, and lightweight.

A standard file structure is utilized to ensure that the software architecture is consistently able to locate and access the files necessary for its operation from predictable locations irrespective of the underlying hardware on which it executes or the storage medium on which these files are located. Access to these files by the users of the system is also made more convenient. The file structure designed for the purposes of the software architecture consists of four directories as well as up to four different types of files. These directories and files are described below.

- **Template Class Directory**—The *template class directory* amss/algo is the directory in which the Java classes, termed the *logical module template classes*, are placed. These classes provide the platform-independent intelligence that enables connected TIMs to collaborate with each other and operate as a logical entity.
- **Module TEDS Directory**—The *module TEDS (Transducer Electronic Data Sheet) directory* teds consists of one or more text files termed *module TEDS*, each possessing the extension .mod. These files identify and describe the characteristics and digital data format of the transducers associated with a particular physical TIM in the form of a list of *property-value pairs*. The usage of a text format instead of a binary format enables the TEDS to be specified in a easily human-understandable and easily modified form.
- **Template TEDS Directory**—The *template TEDS directory* tmpl consists of zero or more text files termed *template TEDS*, each associated with one template class, that identify and describe the characteristics of a combination of collaborating TIMs known as a *logical module*. Template TEDS also specify the various *roles* that may be fulfilled by a particular class of TIMs within the logical entity. Template TEDS are specified using the same format as module TEDS and possess the same .mod extension.
- **ARC4 Key File**—The *ARC4 key file* key.rc4 stores the variable-length *key* required by the *Alleged Rivest Cipher 4* (ARC4) cryptographic stream cipher [24] utilized by the software architecture for the secure transmission of packets. Modules are only able to communicate with others that are utilizing the same key.
- **Network Identifier File**—The *network identifier file* net.id stores the 5-byte *network identifier* used to indicate that a particular TIM is a member of a network of TIMs possessing the same network identifier. Packet transmissions from modules with different network identifiers are completely ignored, thus reducing packet processing overhead.

## 4. Communication Layer

The purpose of the *communication layer* is to provide *logical link control* in the form of a secure, reliable, connection-oriented service; *medium access control* to prevent channel access conflicts; a mechanism for *time synchronization* between modules; and *wireless security* through the encryption of transmitted data, facilitated by a cryptographic stream cipher. The communication layer accepts *messages* from the middleware layer and splits them into discrete *packets*, which are then encrypted and transmitted through the wireless transceiver driver. Conversely, the communication layer also accepts and decrypts incoming packets from the wireless transceiver driver, merges them into messages if necessary, and passes them to the middleware layer. The communication layer also implements a wired protocol that facilitates the direct transmission of data through the faces of physically connected TIMs.

### 4.1. Logical Link Control

Wireless communication tends to be very unreliable in the absence of an error correction mechanism, mainly due to the regular interference encountered by radio waves during their propagation. Therefore, a reliable, connection-oriented service must be provided within the communication layer to ensure that transmitted packets arrive error-free and in the correct order. This service is facilitated in the form of a *Positive Acknowledgement with Retransmission* (PAR) data link protocol, which is partially implemented within the nRF24L01 transceiver hardware under the Enhanced ShockBurst^™^ feature set [19].

In this protocol, each packet that requires guaranteed transmission is automatically acknowledged by the receiving module through the use of an Enhanced ShockBurst^™^ *acknowledgement packet* (ACK). Packets for which an acknowledgement is not received are automatically retransmitted. A message which is larger than the 96-bit *maximum transmission unit* (MTU) defined by the communication layer (see Section 4.5) is fragmented into multiple packets before transmission.

### 4.2. Medium Access Control

Since a modular sensing system will normally be comprised of a number of collaborating modules, a *Medium Access Control* (MAC) protocol needs to be provided within the communication layer to share the single multi-access broadcast channel among the many contenders competing for control of the medium. A MAC protocol may generally be described as being either a *static allocation* protocol or a *dynamic allocation* protocol. In static allocation protocols, channel bandwidth is divided into equally sized portions, with each portion allocated to one transmitting device. In dynamic allocation protocols, channel bandwidth is allocated to each transmitting device on an as-needed basis. The following MAC protocols were considered for use in the software architecture:

The MAC protocol utilized in the modular sensing system software architecture is a dynamic allocation protocol based on MACAW. The MAC protocol was derived from MACAW because its acknowledgement and carrier sensing features are already implemented in hardware within the nRF24L01 transceiver, facilitating improved performance. In addition, MACAW does not depend on global time synchronization between contenders for the medium in order to operate reliably, which is a weakness of the static allocation protocol *TDMA* (Time Division Multiple Access) [15] and the dynamic allocation protocol *ALOHA* [15]. This is important because much of the communication layer is implemented in a concurrently executing task running on a pre-emptive real-time operating system (see Section 3.4). As a result, the task executes at unpredictable intervals, making reliable global synchronization very difficult to achieve.

### 4.3. Time Synchronization

The timers used within each module to generate and compare timestamps need to be regularly synchronized during operation. Regular synchronization is necessary since the resonant frequency of the crystal oscillator that controls the local time of each module may be slightly different from its rated value, or may shift slightly over time. These variations in resonant frequency result in varying degrees of *clock drift* between module clocks, and in turn cause a loss of synchronization between the local times of each module. System reliability is therefore reduced, since the reported time of occurrence of an event by a particular module may not necessarily be accurate with respect to the local time of another module.

**Figure 5.**
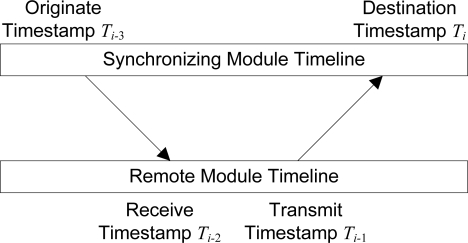
Time synchronization packet exchange.

The protocol used for time synchronization is a distributed protocol based on the *Simple Network Time Protocol* (SNTP) developed by Mills [25], which is a subset of the *Network Time Protocol* (NTP) also developed by Mills [26]. Both SNTP and NTP are standard, well-known protocols widely used to synchronize computer clocks over the Internet. As described in [25], and shown in Figure 5, four 64-bit timestamps are generated in the synchronization procedure, each relative to the clock of the module on which it was taken. The *roundtrip delay δ* of exchanged packets, and the signed *clock offset θ* which is added to the local clock of the synchronizing module, are then accurately calculated by the synchronizing module using Equations 1 and 2, derived by Mills [26].

(1) $$\delta =(T_{i}-T_{i-3})-(T_{i-1}-T_{i-2})$$

(2) $$\theta =\frac{\left(T_{i-2}-T_{i-3})+(T_{i-1}-T_{i}\right)}{2}$$

### 4.4. Wireless Security

Unlike wired transmission mediums such as twisted-pair Ethernet cables, wireless transmission is inherently insecure since the modulated radio signals used for data transmission are easily intercepted by any individual possessing a tuner of the appropriate frequency. Wireless security is provided within the communication layer in the form of *encryption* in order to provide confidentiality when privacy of information pertaining to the identification of TIMs and their collected data (which is frequently transmitted wirelessly between modules) is required.

Two common cryptographic algorithms that facilitate information security are *stream ciphers* and *block ciphers*. In a stream cipher, the bits comprising information to be transmitted are combined with a pseudorandom *keystream* of *cipher bits* through the use of the *exclusive-or* (XOR) logical operation. The cipher bit stream varies with a *key* used to initialize the algorithm. In a *block cipher*, information is processed in fixed-length groups of bits known as *blocks*, which are typically much larger than one byte, thus requiring the length of the information provided for encrypting to be a multiple of the block size. An pair of complementary *transformation functions* are used for encryption and decryption, the behaviour of which is unique to a supplied *key*, and are applied to information blocks to produce encrypted blocks, and to encrypted blocks to produce information blocks, respectively. Block ciphers are typically slower than stream ciphers and often require the usage of more memory during their operation; however, block ciphers often facilitate the creation of encryption algorithms that provide a greater degree of security compared to stream ciphers.

All packets generated for transmission by the software architecture are encrypted using the *Alleged Rivest Cipher 4* (ARC4) [24] encryption algorithm due to its straightforward implementation, excellent speed, minimal memory usage, and relatively strong security. These criteria are important due to the resource-constrained hardware present in the TIMs.

### 4.5. Packet Format

**Figure 6.**
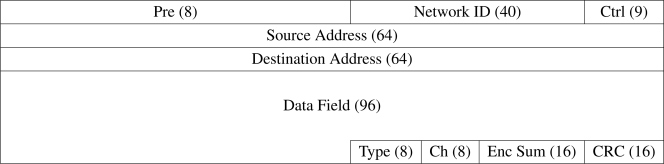
Communication layer packet format (field sizes in bits).

Data is transferred to and from the transceiver driver in 329-bit *packets*. The packet format, shown in Figure 6, is designed to be compatible with that of the nRF24L01 transceiver, which manages the *preamble*, *network identifier*, *packet control field*, and *CRC checksum* fields within its firmware. The 32-byte payload field defined within the nRF24L01 packet format is sub-divided into smaller fields for the purposes of the software architecture.

The *preamble* is an alternating binary pattern facilitating low-level synchronization between transmitters. The *network identifier* identifies the overall network of modules from which incoming packets are accepted. The *packet control field* facilitates detection of packet retransmissions and also indicates if the packet requires acknowledgement. The *source address* and *destination address* identifies the physical or logical module that transmitted or should receive the packet respectively. The *data field* contains the data transmitted within the packet, and may be further sub-divided into *parameter fields*. The *packet type* dictates how the data field should be interpreted. The *packet channel* indicates the channel used for transmission. The *encryption checksum* is a simple checksum used to verify decrypted packets. The *Cyclic Redundancy Check checksum* is used to detect transmission errors.

### 4.6. Channels and Packet Types

The nRF24L01 transceiver is able to transmit and receive packets on any one of up to 125 distinct radio frequency channels at a time, one of which is reserved by the software architecture for use as a *control channel*. All modules listen to the control channel by default when not transmitting data, and each module can detect the presence of others in its vicinity by listening for packet transmission activity on the channel. At regular intervals, each module broadcasts *PRE* (presence) packets indicating their continued presence in the sensing system, and *MEM* (member) packets indicating their continued presence in any *logical modules* of which it is a member. Time synchronization is also carried out on the control channel at regular intervals through the use of *SYQ* (synchronization query) and *SYR* (synchronization response) packets. Due to the relatively long intervals between packet transmissions on the control channel (see Section 4.7) in comparison to the time required to transmit individual packets, the communication overhead incurred by the various protocols that utilize the control channel is negligible.

The other 124 channels are utilized as *data channels*. Upon successful reservation of a data channel through the use of the *RTS* (Request To Send) and *CTS* (Clear To Send) *medium allocation packets*, the transmitting and receiving modules switch to the agreed channel and carry out the transmission of *DAT* (data) packets comprising *messages*. Lengthy transmissions may occur simultaneously on different channels without interference.

### 4.7. Network Communication Task

**Figure 7.**
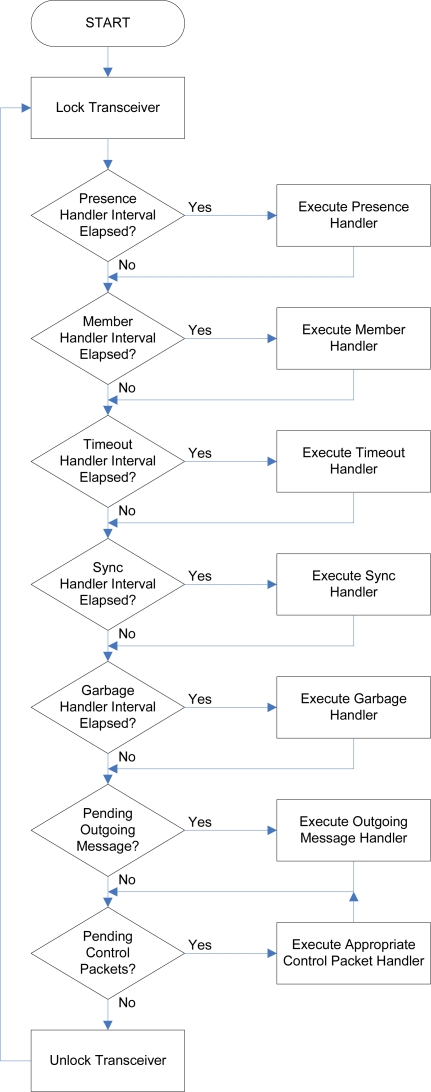
Network communication task operation.

The *network communication task* is started upon initialization of the software architecture and runs continuously and concurrently with all other tasks in the system. The general operation of the network communication task is depicted in Figure 7. The network communication task is required to carry out a number of functions, each of which is performed by a specific *handler*. These handlers are described as follows:

#### 4.7.1. Presence Handler

Transmits presence packets every two to five seconds for all the physical modules represented on a TIM, as well as for the logical modules for which the TIM is the *primary* module. The primary module possesses the lowest address among the members of the logical entity and is responsible for transmitting and processing its messages.

#### 4.7.2. Member Handler

Transmits member packets every two to five seconds indicating which the logical module entities the physical and logical modules represented on a TIM are a member of. The roles in the logical entities fulfilled by each module is indicated within these packets.

#### 4.7.3. Timeout Handler

Used to detect modules that have left the environment. Presence and member packets received by a TIM from another module within the environment are assigned a local counter on the TIM. This counter is decremented every second by the timeout handler and reset when another presence or member packet is received from the remote module. If the counter expires, the remote module is deemed to have left the environment, or the associated logical module, respectively.

#### 4.7.4. Synchronization Handler

Synchronizes the local clock with those of nearby modules every one to two minutes. Synchronization is only performed if a module with a lower *synchronization level* (indicated within the transmitted presence packets) is found in the environment, or a module with an equivalent synchronization level, but a lower address, is found. Upon synchronization, the synchronization level of the synchronized module is subsequently updated to be one more than that of the module to which it was synchronized, up to a maximum of 255. Interconnect, standard, and administration modules are initialized with synchronization levels of 255, 254, and 1 respectively, where lower synchronization levels correspond to more accurate clock references.

#### 4.7.5. Garbage Handler

Reclaims memory allocated by completed tasks every two seconds. This is necessary since tasks cannot deallocate their stack and heap space on their own upon completing their execution.

#### 4.7.6. Outgoing Message Handler

Transmit a single outgoing message, if any is pending, on each loop iteration.

#### 4.7.7. Control Packet Handler

Handle up to five pending control packets, if any were received, on each loop iteration. If an *RTS* packet is pending, handshaking is carried out and the message reception mechanism is invoked.

## 5. Face Connectivity

**Figure 8.**
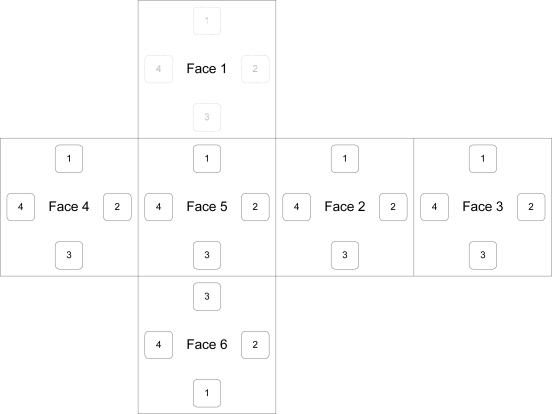
Two-dimensional face and contact identifier layout view.

Provided within the communication layer is a wired protocol that facilitates the direct transmission of identification data through the faces of physically connected TIMs, as well as the determination of the relative angular offset between the connected TIMs. The operation of this protocol depends on the electrical contacts present on the four clips located on five of the six faces of a TIM. In order to facilitate the detection of the relative angular offset between two connected TIMs, the TIM faces as well as the electrical contacts located on them are each assigned an *identifier*. Each TIM face is assigned an identifier from 1 to 6, while each face contact is assigned an identifier from 1 to 4, as depicted in Figure 8.

### 5.1. Face Identification Packet Format

**Figure 9.**
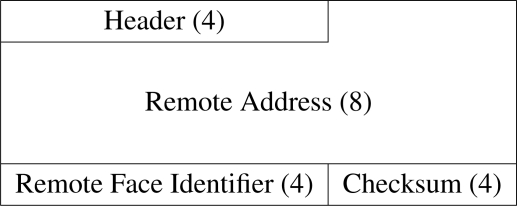
Face identification packet format (field sizes in bytes).

Face identification information is transferred between faces in the form of 20-byte *face identification packets*, the format of which is depicted in Figure 9. The information transferred within these packets reveals of the address of the physically connected remote module as well as the identifier of the connected face through which the packet was received. Examination of the local contact through which the packet is received also enables the relative angular offset between the connected TIMs to be determined.

**Figure 10.**
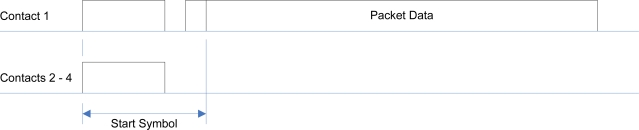
Face contact transmission signals.

Face identification packets are transmitted on each face every five to ten seconds, indicating the continued presence of a physical connection on the respective face to another module. Data signals are transmitted in a format similar to that of *RS-232* [27], in that transmissions are composed of a *start symbol* followed by an asynchronously timed series of bits, as depicted in Figure 10. An incoming packet is indicated by high logic levels being detected on all of the contacts on the face. The contact on which data will be transmitted is detected through examination of the start symbol.

### 5.2. Face Communication Task

**Figure 11.**
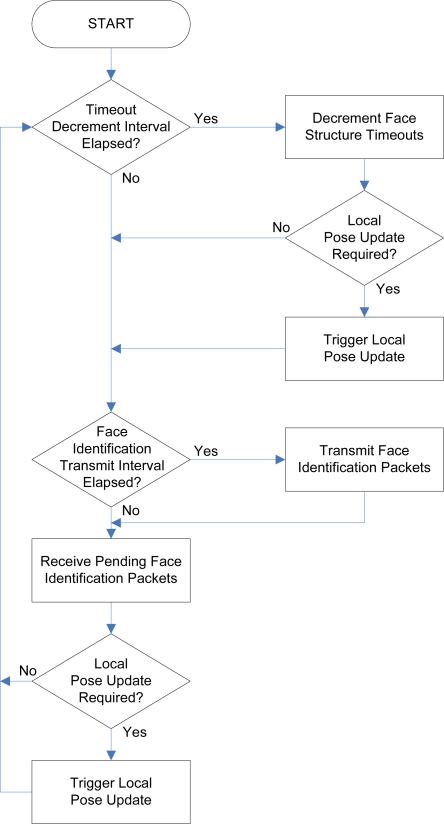
Face communication task operation.

Like the network communication task, the *face communication task* is started upon initialization of the software architecture and runs continuously and concurrently with all other tasks in the system. The general operation of the face communication task is depicted in Figure 11. The face communication task is required to perform the following operations:

- Decrement the five face identification packet *timeout counters*, which are stored in the five data structures representing the state of each connectable face, every second. If a connected module is discovered to be disconnected or unresponsive due to the expiry of one of the counters, update the local *pose* (represented by a 4 × 4 matrix of floating point values) if necessary.
- Transmit face identification packets on each face indicating the address of the module and the respective face identifier every five to ten seconds.
- Receive pending face identification packets, if any, from any remote modules connected to each face. If a newly connected module is discovered, update of the local pose if necessary.

## 6. Middleware Layer

The purpose of the *middleware layer* is to facilitate interoperability between the various TIMs in a modular sensing system. The term *middleware* refers to software and services that simplify connectivity between software components running on distinct and possibly heterogeneous devices, in turn simplifying the deployment of distributed applications. At the middleware layer in this software architecture, the *application programming interface* (API) for physical and logical modules is defined, which is comprised of a variety of *service functions*. Service functions are the interface through which TIMs, whether homogenous or heterogeneous, request services from, and information about, each other. Data is transferred between TIMs in the form of variable-length *messages*.

### 6.1. Middleware Classifications

**Figure 12.**
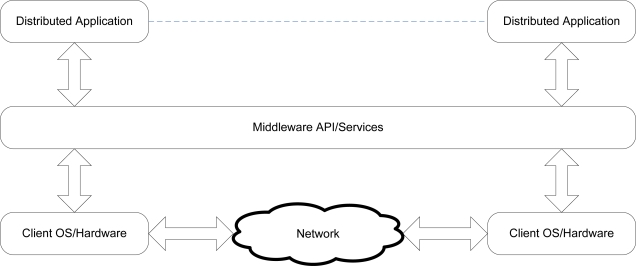
Middleware operation block diagram.

Middleware implementations lie between the operating system and the distributed application as shown in Figure 12, and are classified as being either *synchronous* or *asynchronous*. Synchronous systems require that each middleware request be carried out to completion before any further requests are processed. As a result, multiple threads of execution are necessary for parallelism to occur. Conversely, asynchronous systems allow multiple requests to be issued without requiring the prior completion of any single request. However, responses are not guaranteed to be processed in order within any single thread of execution.

The middleware layer of the modular sensing system software architecture is based on the asynchronous *Message-Oriented Middleware* (MOM) [28] implementation. In MOM implementations, *messages* are passed between devices on a network. Messages received by a client are stored in a *message queue* until they are able to be processed. The client may continue processing other data while incoming messages are enqueued. For the purposes of the software architecture, a synchronous message transmission mechanism was also implemented.

### 6.2. Message Format

**Figure 13.**
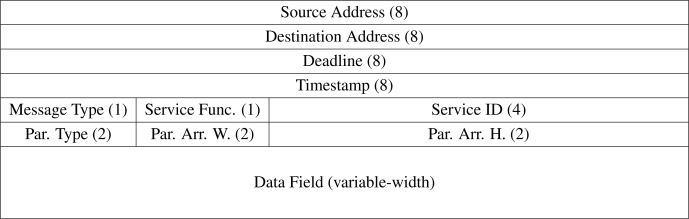
Middleware layer message format (field sizes in bytes).

A middleware layer *message* consists of a 44-byte header, followed by a single variable-length block containing the data to be transferred in the message, as shown in Figure 13. Modules request data and services from other modules by issuing *service call* messages. The requested data or the results of the service call are transmitted back to the caller in the form of *return* messages, either synchronously or asynchronously as demanded by the template class running on the caller.

The *source address* and *destination address* identifies the physical or logical module that transmitted or should receive the message respectively. If the message is call to a service function, the *deadline* indicates the time at or before which the call should be completed. The *timestamp* indicates the time at which a particular message was enqueued for transmission, or the time at which a particular event occurred. The *message type* indicates how the message should be processed. The *service function* indicates what service function should be or was invoked. The *service identifier* uniquely identifies a service call message and its associated return message. The *parameter type* indicates the type of data supplied as parameters within the *data field*. These parameters are organized into a two-dimensional array of fixed-sized elements, the dimensions of which is indicated by the *parameter array width* and *parameter array height*. These elements may be numerical values, strings, or raw binary data.

### 6.3. Service Functions and Service Calls

**Figure 14.**
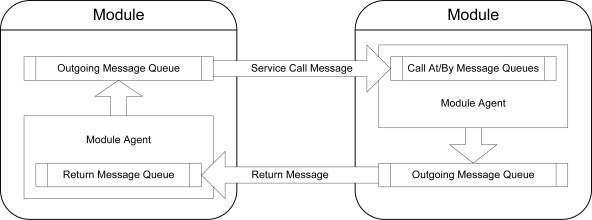
Service call operation.

*Service functions* enable modules to provide services to and exchange information with each other. These functions may be invoked automatically by other modules within the network, or manually through an administration module. The call/reply mechanism used during the invocation and processing of service functions, known as a *service call*, is based on the standard, widely used *Remote Procedure Call* (RPC) protocol [29]. As seen in Figure 14, a service call is invoked by a module through the placement of a *Call By* or *Call At* message in the single outgoing message queue present on a TIM. This message specifies the service function type and contains the relevant parameters to the function.

Once the transmitted message is received by the destination module, it is placed in the appropriate *Call By* or *Call At* incoming message queue. The message is processed by or at the specified deadline respectively, and a *Return* message containing the results of the service call is placed in the outgoing message queue for transmission to the invoking module. This return message possesses the same *service identifier* as the call message, enabling it to be identified by the calling module as the results of the service call even if unrelated return messages are received from other modules before the call is completed. The return message may also contain a *status constant* instead of data, indicating the success or failure of the service call.

Service calls are issued either *synchronously* or *asynchronously* to a remote module. A synchronous service call causes the real-time operating system to suspend the calling task until the corresponding return message is received from the remote module or the service call times out, while during an asynchronous service call the task is allowed to concurrently continue execution while the call is being processed. The various service functions defined by the software architecture that enable the state and properties of a module to be obtained or modified are as follows:

- **Get**—Obtains the value or state of the transducer associated with the target TIM.
- **Set**—Modifies the state of the transducer associated with the target TIM.
- **Append**—Adds to the state of the transducer associated with the target TIM instead of changing it directly.
- **Reset**—Resets the state of the transducer associated with the target TIM to its default value.
- **Get TEDS**—Retrieves from the TEDS property list of the target module the value associated with a provided property name.
- **Get Pose**—Obtains a copy of the *pose matrix* (see Section 8.3) representing the pose (position and orientation) of a TIM.
- **Update Pose**—Upon connection to a tree of physically connected TIMs, transforms the coordinate space of the pose matrix of the target TIM into that of the *pose base* (see Section 8.3) of the tree.
- **Lock**—Locks a TIM, enabling a sequence of modification operations (*Set*, *Append*, and *Reset*) issued by another module to be performed atomically.
- **Unlock**—Unlocks a previously locked module.
- **Join**—Incorporates a TIM into a new or existing logical entity.

### 6.4. Module Message Handler Task

**Figure 15.**
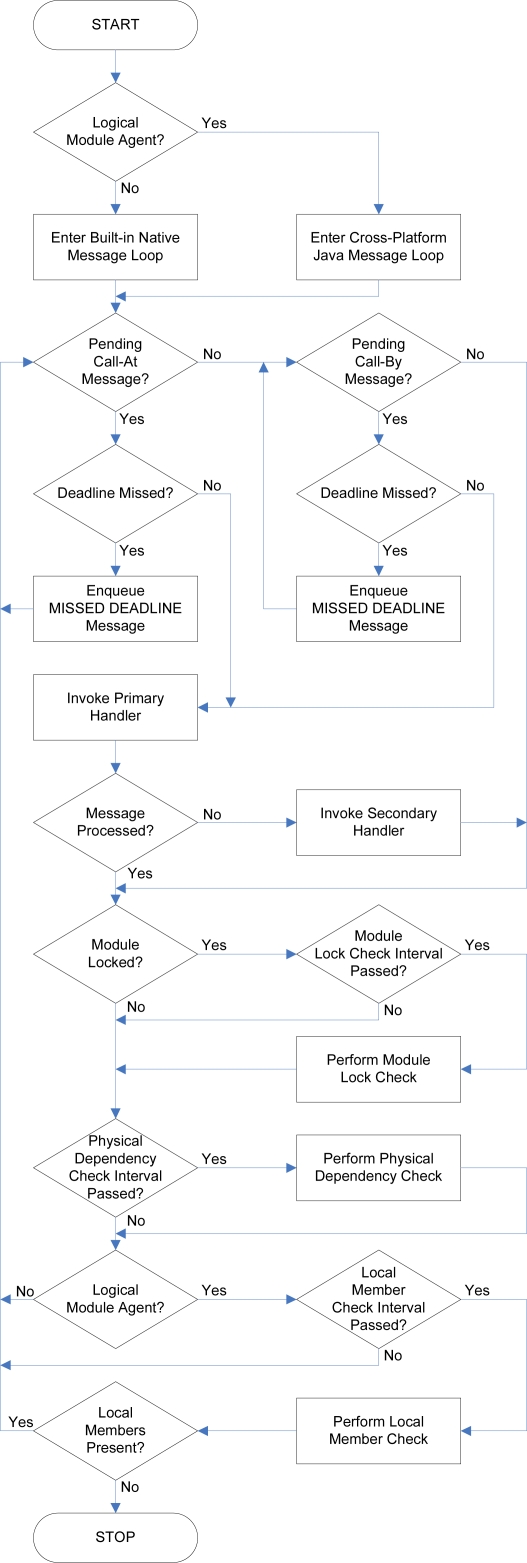
Module message handler task operation.

Each combination of the *state* of a module and an associated *module message handler task* is termed a *module agent*. The module message handler task continuously examines and updates the queues and status of its associated module agent and generates messages in response to received messages and other events that occur within its environment. This intelligence is either provided natively within the module firmware or by platform-independent template classes. Through the use of a real-time operating system within the software architecture, multiple module message handler tasks may execute concurrently on a single TIM, each receiving and transmitting messages in real time. The general operation of a module message handler task is depicted in Figure 15. The module message handler task is required to carry out the following operations:

- Obtain the next service call to be processed, if any, from the *Call At* and *Call By* message queues. Call At and Call By messages are inserted into their respective queues by deadline, but since Call At messages need to be checked more regularly for a deadline match, using separate queues ensures that the next Call At message is always accessible within a single operation. This might not always be possible if a single queue was used for both message types.
- Process the service call by invoking the *primary handler*, and if necessary, the *secondary handler*. For physical module agents, the primary handler is the relevant native driver function for the transducer. For logical module agents, the primary handler is found within its associated cross-platform template class. The primary handler is expected to process service calls that obtain or modify the value and state of its associated transducer. The secondary handler, defined within template classes, is invoked to process the service call if the primary handler provides no suitable implementation for handling it. The secondary handler also processes service calls that obtain or modify the pose and properties of a module. If the service call is still not handled after invocation of the secondary handler, it is deemed to be invalid and is dropped.
- Perform various *status checks* that ensure the integrity of the module agent is maintained throughout changes to the environment in which it executes. The checks include ensuring that the module is not perpetually *locked* while executing atomic service calls; ensuring that modules composing a logical entity formed using physical connections are still physically connected; and ensuring that the remote members of a logical entity of which a module agent is a member are still present within the environment.

## 7. Virtual Machine

To facilitate the adaptability and reconfigurability of a group of physically or wirelessly collaborating heterogeneous TIMs, a dynamic reprogramming mechanism is necessary that allows the TIMs to automatically source, load, and execute platform-independent logical module template algorithms at run-time. In this software architecture, this functionality is facilitated through the use of a *virtual machine* (VM), which is a program that interprets and executes high-level, hardware-independent abstract *bytecodes*. Each bytecode is a sequence of one or more bytes that represents an instruction to be executed by the VM. Algorithms defined using these bytecodes are therefore completely decoupled from the underlying hardware architecture on which they execute, and may therefore be specified once and then used, without recompilation, in the dynamic reprogramming of a variety of heterogeneous modules and hardware architectures as application requirements change. As a result, these algorithms are also easy to create, debug, and maintain.

### 7.1. Dynamic Reprogramming Mechanism

The virtual machine dynamic reprogramming mechanism implemented within this software architecture is a lightweight implementation of Sun Microsystems’ *Java Virtual Machine* (JVM) [21,30], supported by an architecture-specific standard class library. The use of a Java-based virtual machine provides the software architecture with a powerful and well-established platform in which hardware-independent algorithms may be specified. Due to the widespread adoption of and compiler support for the JVM, there is great flexibility in the choice of algorithm specification language. At the core of the virtual machine is a continuously executing bytecode interpretation loop that implements a *fetch, decode, execute* cycle similar to that found in modern microprocessors. A disadvantage of using a virtual machine for dynamic reprogramming mechanisms is the moderate execution overhead incurred during bytecode interpretation. However, this overhead is negligible on sufficiently fast hardware, such as that utilized in this software architecture.

Upon the creation of a logical module agent, an appropriate platform-independent *logical module template class* from the *template class directory* amss/algo is loaded to provide the intelligence necessary for each member TIM to function as part of the logical entity. The standard entry point for this class is the method main, as is normal for Java classes that are intended to be executed. However, unlike typical executable Java classes that accept a String array as a parameter to the main method, logical module template classes accept a Module class reference specific to the software architecture. This reference provides an interface through which the behaviour of the members of the logical module may be controlled within the template class.

### 7.2. Standard Class Library

The software architecture provides a standard collection of classes and methods, grouped into *packages*, designed specifically for use within template classes. These classes are not physically present on the local storage of the modules; rather, invocations of the methods within these classes, which occur very frequently, are caught and handled natively at runtime. This results in minimal flash memory and RAM usage, and also substantially increases performance due to the removal of interpretive overhead for these classes.

Due to flash memory and RAM constraints, the extensive standard class library provided within a full Java implementation is not present in its entirety. Provided instead is a very lightweight and useful subset of the standard class library as well as architecture-specific classes that encapsulate the functionality necessary to support the collaboration of a group of TIMs. These classes are outlined in the following two subsections.

### 7.3. The java.lang Package

The official Java class library is comprised of numerous packages and classes that provide a base for the development of Java applications. One of the most important of these packages is java.lang, which provides critical functionality such as mathematical operations and string processing. A subset of the complete java.lang package was included within the standard class library of the software architecture, to provide these two critical features. The two constituent classes are outlined below:

- **Math**—The class java.lang.Math contains methods that facilitate the calculation of various arithmetic and trigonometric operations. It also defines the mathematical constants *e* and *π*.
- **String**—The class java.lang.String is used to represent an immutable string of characters, and contains methods that facilitate a variety of commonly used string comparisons and operations.

### 7.4. The amss.system Package

**Figure 16.**
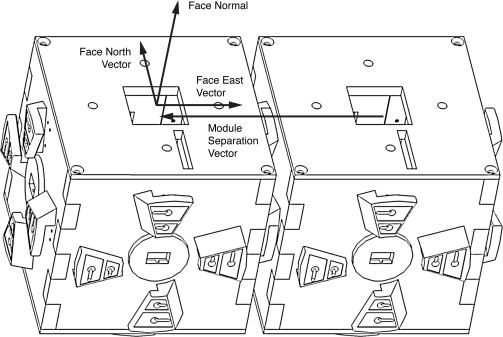
Module pose vectors.

In addition to the java.lang package, a package of classes unique to the software architecture is provided to support and provide an interface to the core functionality of the architecture. This package, amss.system, consists of the five classes outlined below:

- **AMSS**—The class amss.system.AMSS contains methods and constants that support various system-level operations provided by the software architecture, including explicit garbage collection and atomic operations.
- **Message**—The class amss.system.Message contains methods and constants that facilitate performing operations on and obtaining data from messages.
- **Module**—The class amss.system.Module contains methods that facilitate performing operations on and obtaining information about physical and logical modules, such as querying the state and properties of a module, determining the number of matched modules to each role within a logical entity, and issuing service calls to and retrieving results from matched members.
- **Pose**—The class amss.system.Pose contains methods that facilitate the acquisition of five *pose vectors* that are derived from the matrices representing the poses of one or a pair of modules. These pose vectors are utilized within template classes to facilitate the determination of the relative positions between any two indirectly or directly connected modules in a logical entity, irrespective of their orientations. The first vector represents the absolute position of a module; the other four vectors are depicted in Figure 16.
- **Vector3D**—The class amss.system.Vector3D contains methods that facilitate operations on three-dimensional vector quantities.

## 8. Composition Layer

At the composition layer, *logical module template algorithms* are loaded and executed that enable a group of TIMs to collaborate and behave as a logical entity known as a *logical module*. The intelligence needed to facilitate module collaboration is encompassed within a Java class that is interpreted by the virtual machine, and is accompanied by a *logical module template TEDS* that describes the standard characteristics of the composite logical module entity. Within each template TEDS, various *role descriptors* are also defined. A role descriptor represents the characteristics required of a particular module agent within the environment to satisfy a particular behavioural *role* within a logical entity.

Each module agent within a sensing system continually tests the others in its environment against the roles defined within its locally stored logical module template TEDS in order to locate a match. If a module agent is found that is capable of providing the sensing or actuation behaviour outlined by at least one of the specified roles, the matched agent will be assimilated into an existing or a newly created logical entity containing the matched role. Physically connected TIMs within a logical entity will also intelligently relay their position and orientation to each other, ensuring that all the member TIMs comprising the logical entity possess a representation of their position and orientation that is relative to the pose of one of the members, designated the *pose base*.

### 8.1. Template and Role Matching

Creation of new logical module entities or the addition of module agents to existing logical modules is facilitated through the matching of the various *roles* defined in a logical module template TEDS. Each TIM in a sensing system observes the *presence* (PRE) packets being transmitted by the other modules within its environment, which are first tested against the logical module template representations already loaded into memory, and then against the various template TEDS specifications present within the local template TEDS directory of each TIM. As previously stated, each presence packet contains various fields that reveal the capabilities of the module that transmitted it. Thus, matches against templates represent suitable candidate modules that may be used in the formation or augmentation of logical modules.

Template matches are only attempted by a TIM when new modules are detected within its environment, or if the module is physically connected to or disconnected from another. Only physical connections and disconnections trigger template TEDS directory searches, since frequently searching the template TEDS directory for matches is expensive in terms of processing time and memory usage, especially when the directory consists of numerous TEDS specifications. In order to prevent infinitely recursive matching, a logical module is not allowed to match itself. Logical module creation proceeds when the assignment constraints for every role in its associated template is satisfied, in which a *join* message is issued by the *primary* module of the logical entity to each of the matched candidate module agents. The primary module possesses the lowest address of all detected member modules comprising the logical entity and is responsible for managing its operation. A *module message handler task* (see Section 6.4) associated with the logical entity is created on each member module such that during the times in which any member module assumes the position of primary member, that module is capable of processing messages addressed to the logical entity.

The issued *join* messages provide information to each candidate module agent indicating the location of or containing the logical module template TEDS and template class it should load, identifying the matched role it should perform, as well as identifying whether membership within the logical entity depends on the candidate module agent being physically connected to other members of the logical entity. Allowing the data comprising a template TEDS and template class to be transmitted within a *join* message enables the library of template TEDS specifications and processing algorithms available to the modules within a particular sensing system to be distributed and remain up to date in an automatic, peer-to-peer fashion, without requiring user intervention. For newly created logical modules a random 64-bit *logical module address* not already present within the environment is generated and assigned to it. The most significant bit of this address, termed the *logical module bit*, is always set, and differentiates logical entities from standard module agents.

### 8.2. Transducer Composition

**Figure 17.**
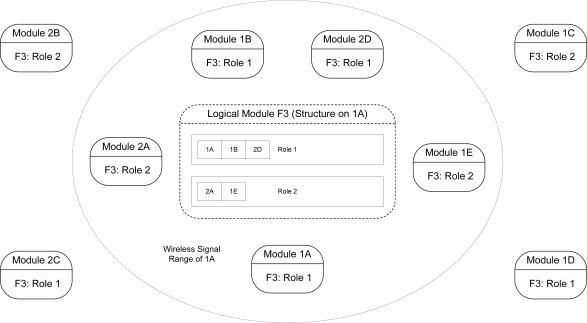
Logical module operation block diagram.

The mechanism behind transducer composition in logical modules is illustrated in Figure 17, using the example of a logical module possessing two defined roles. Each role within the memory representation of a logical module possesses a *role environment list*, which stores *member* (MEM) packets corresponding to the currently detectable module agents within the environment that were assigned to the role after having been issued a *join* request.

As shown in Figure 17, the logical module F3 (8-bit addresses are depicted for simplicity), which has a representation present in memory on all its members, consists of two roles. Module 1A is the *primary member* due its possession of the lowest address among the members of the logical entity. Therefore 1A currently processes messages transmitted to the logical module, and also generates messages on behalf of the logical entity. Since only four of the eight other modules that comprise F3 are within range of 1A, the regularly transmitted member packets of these four modules are the only ones that are detectable by F3. Thus, from the perspective of 1A, only five modules, including itself, are currently available to fulfil the specific roles within F3 that they were matched and assigned to.

Readings from physical and logical modules are acquired through *Get* service calls. Within a call to the *Get* service call handler for the logical module F3, the *role environment counts* for each of its defined roles are determined. The role environment count is the number of detected members in the environment that satisfy a particular role. The role environment counts are then utilized within the template class providing the intelligence for F3 to acquire transducer readings from all the member modules within each role through the invocation of a number of *Get* service calls. These readings are then stored or processed on the fly to produce a composite reading that is returned to the invoking module. If other logical modules are assigned to fulfil roles within the logical module, the composition process is recursively invoked on each member logical module until the composed readings produced by each are generated and returned up the tree of logical modules. Combined with the ability to dynamically determine role environment counts, service functions provide convenient access to the services of the member modules available to a particular role without necessarily knowing how many members are accessible or their addresses.

### 8.3. Pose Representation and Theory

The *pose* (position and orientation) of each TIM in a modular sensing system is represented locally on each TIM in the form of a 4 × 4 *pose matrix* **P**. Other popular methods as described in [31] of representing orientation itself include *Euler angles* and *quaternions*. A matrix is chosen to represent position and orientation rather than Euler angles and quaternions because among these, only a matrix can provide a unique representation of a given orientation. Euler angles in particular are susceptible to the phenomenon of *gimbal lock*, where a degree of freedom may occasionally be lost due to a pitch rotation of 90 degrees causing the roll and yaw rotations to effectively occur about the same axis. Matrices also facilitate the convenient representation of position and orientation as an atomic, combined entity, enabling operations to be performed on both a position and its associated orientation simultaneously. In addition, to facilitate transformations between coordinate spaces, representations such as Euler angles and quaternions must, in any case, be converted to matrix representations. The data stored within a pose matrix is as shown in Equation 3:

(3) $$P=\left[\begin{array}{cccc} {\mathrm{ax}}_{x} & {\mathrm{ay}}_{x} & {\mathrm{az}}_{x} & p_{x}\\ {\mathrm{ax}}_{y} & {\mathrm{ay}}_{y} & {\mathrm{az}}_{y} & p_{y}\\ {\mathrm{ax}}_{z} & {\mathrm{ay}}_{z} & {\mathrm{az}}_{z} & p_{z}\\ 0 & 0 & 0 & 1 \end{array}\right]$$

**Figure 18.**
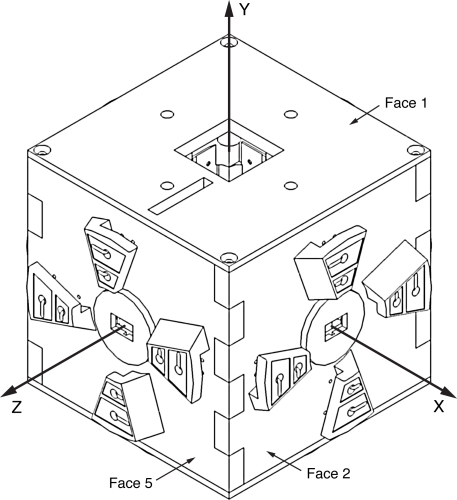
Standard TIM object coordinate space.

Each column within the pose matrix represents a three-dimensional geometric vector, defined relative to the cardinal axes within a right-handed coordinate system. The first three columns of the pose matrix, *ax*, *ay*, and *az*, respectively represent the *x*, *y*, and *z* axes defining the *object coordinate space* of the TIM, depicted in Figure 18. The subscripts *x*, *y*, and *z* of the terms *ax*, *ay*, and *az* represent the *x*, *y*, and *z* components of the axis vectors defined relative to the cardinal axes within the coordinate system. The fourth column represents the absolute position in centimetres (cm) of the TIM (more specifically, the *origin* of its object coordinate space) relative to the cardinal axes and within the object coordinate space of the *pose base*, described within the following paragraph. The matrix representation chosen facilitates the transformation of one TIM coordinate space into another to be performed through consecutive pose matrix multiplications applied from right to left.

The pose matrix of each TIM is set to the identity matrix upon initialization, resulting in the axis vectors being identical to the cardinal axes within the coordinate system, and the position being identical to the origin. However, within a TEDS specification, the initial pose may be overridden through the specification of initial rotations about the cardinal axes, and an initial translation from the origin. These user-specified transformations may be derived from measurements obtained using external measurement systems. The transformations are thereafter immediately applied to the identity pose matrix when the TEDS is loaded.

Within a logical module comprised of physically connected TIMs, the pose of all member modules must be defined relative to the object coordinate space of a single member module defined as the *pose base*. The use of a pose base allows each member module to access the pose of any other physically connected member knowing that the pose matrix returned will be within the same coordinate space as the locally represented pose. This may be done even if the TIMs are indirectly connected through any number of physically connected members. The transformation of the pose matrix coordinate space of member TIM **A** into that of member TIM **B** is facilitated through the matrix multiplication shown in Equation 4, applied from right to left:

(4) $$P_{A_{\text{new}}}=P_{B}\times P_{A}$$

### 8.4. Pose Composition

*Pose updates* are triggered whenever physical connections or disconnections between TIMs occur, and are issued through service calls. The local pose matrix of the TIM receiving a pose update service call is transformed through a series of rotations and translations based on pose matrix and face connection information from the remote module provided within the service call. A pose rotation through the cardinal *z*, *y*, and *x* axes of angles *γ*, *β*, and *α* respectively, in that order, followed by a pose translation along these axes of Δ*z*, Δ*y*, and Δ*x* centimetres respectively, is performed through the matrix multiplication shown in Equation 5, applied from right to left:

(5) $$P_{\text{new}}=T\times X_{\text{rot}}\times Y_{\text{rot}}\times Z_{\text{rot}}\times P$$

where:

(6) $$Z_{\text{rot}}=\left[\begin{array}{cccc} \cos\;\gamma & -\sin\;\gamma & 0 & 0\\ \sin\;\gamma & \cos\;\gamma & 0 & 0\\ 0 & 0 & 1 & 0\\ 0 & 0 & 0 & 1 \end{array}\right]$$

(7) $$Y_{\text{rot}}=\left[\begin{array}{cccc} \cos\;\beta & 0 & \sin\;\beta & 0\\ 0 & 1 & 0 & 0\\ -\sin\;\beta & 0 & \cos\;\beta & 0\\ 0 & 0 & 0 & 1 \end{array}\right]$$

(8) $$X_{\text{rot}}=\left[\begin{array}{cccc} 1 & 0 & 0 & 0\\ 0 & \cos\;\alpha & -\sin\;\alpha & 0\\ 0 & \sin\;\alpha & \cos\;\alpha & 0\\ 0 & 0 & 0 & 1 \end{array}\right]$$

(9) $$T=\left[\begin{array}{cccc} 1 & 0 & 0 & \Delta x\\ 0 & 1 & 0 & \Delta y\\ 0 & 0 & 1 & \Delta z\\ 0 & 0 & 0 & 1 \end{array}\right]$$

After the transformations are applied, the local pose of the TIM on which the pose update was invoked will be completely defined in terms of the object coordinate space of the remote TIM. However, the coordinate spaces of all physically connected TIMs must be defined relative to the TIM in the composite entity designated the *pose base*. Since the pose of the remote module would have already been transformed such that it is defined in terms of the coordinate space of the pose base, the local pose may also be brought into the coordinate space of the pose base by multiplying it by the remote pose, as per Equation 4. This operation is valid even if the pose base is only indirectly connected to both the local and remote TIMs, due to the accumulative effect of multiplying transformation matrices in which all previously applied transformations are carried over into successive transformations.

Upon completion of the pose update process, the pose matrices of other TIMs physically connected to the other faces of the updated module will in turn require updating. For each of the physically connected TIMs (except the TIM that issued the initial pose update service call to the updated module), an appropriate pose update service call containing the updated pose matrix and face connection information will be issued.

## 9. Architecture Evaluation

This section presents an evaluation of the behaviour and performance of the software architecture when utilized on actual TIM hardware. This evaluation will be facilitated through two tests, in which select homogeneous and heterogeneous sensors and actuators will be associated with TIMs and connected together. Upon assuming a composite representation, the interactions of the TIMs are then logged locally on the non-volatile storage present on each TIM and examined thereafter.

The first test will be used to evaluate the operation of a logical module in which the constituent TIMs interact entirely through wireless communication. The second test will be used to evaluate the behaviour of a logical module in which the constituent TIMs are physically connected in various orientations, and interact through both wireless communication as well as through their physically connected faces. During these tests, performance criteria that strongly impact the real-world performance of a composite sensing system are considered for each constituent layer of the architecture implemented on top of the real-time operating system, drivers, and module hardware.

### 9.1. Wireless Collaboration Evaluation Setup and Procedure

The purpose of this test is to evaluate the behaviour of a modular sensing system when its constituent TIMs are wirelessly connected. A modular sensing system will be created in which an analog light-dependent resistor (LDR) TIM (designated Module A), a digital accelerometer TIM (designated Module B), and a servo motor TIM (designated Module C) are placed within range of each other. Interfaces to three 5 V adaptors and an RS232 port connected to the personal computer workstation are provided through means of a standard breadboard. These interfaces solely serve the respective purposes of providing a stable voltage to the TIMs and providing a link to their administrative task through which their behaviour may be monitored, and otherwise have no influence of the behaviour of the composite system.

Upon discovering each other, the modules in the sensing system are expected to be capable of utilizing an appropriate template specification and class to automatically form a composite entity that implements a new behaviour. A single composite system should be created that finds and utilizes the average readings of all the available voltage-based sensing TIMs (specifically, the LDR in a potential divider configuration and the accelerometer) in the system to influence the position of all the available rotational actuator TIMs (the servo motor), as per the provided template specification and template class.

To test if the described system behaviour is realized, the accelerometer TIM is physically rotated through five increasing angular degree positions of 0°, 45°, 90°, 135°, and 180° once the formation of a logical module entity is confirmed through the administrative interface. For each accelerometer angle, the LDR TIM is also exposed to high and low levels of ambient light. The angular position assumed by the servo motor TIM is then examined and analyzed in order to determine the correlation between the accelerometer TIM angular positions, LDR TIM ambient light voltage readings, and servo TIM angular positions. The angular position of the servo motor is more easily identified through a black indicator attached to the rotating head.

### 9.2. Wireless Collaboration Results and Analysis

**Figure 19.**
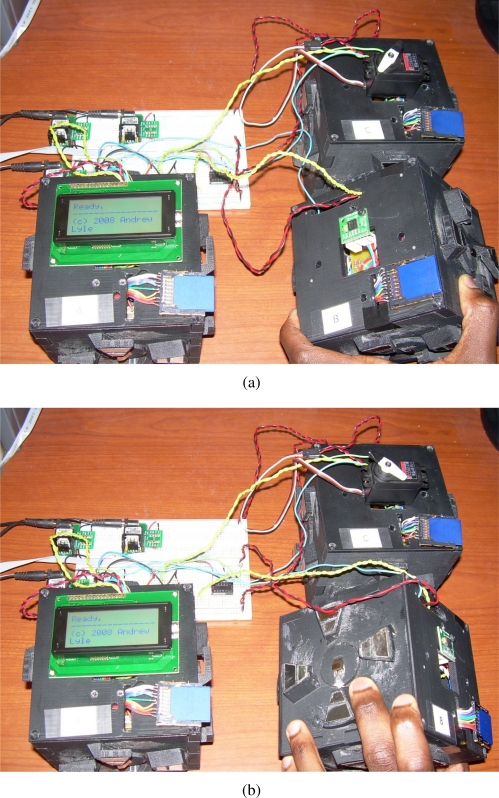
Servo TIM positions for given accelerometer TIM angles. **(a)** Accelerometer TIM angle of 45°. **(b)** Accelerometer TIM angle of 135°.

Figure 19 depicts the behaviour of the composite sensing system formed in the first evaluation setup at angles of 45° and 135°. In the template class executing on primary module C, the readings from all of the acceleration (utilizing solely their *x*-axis readings) and voltage sensing module agents which comprise the logical entity are continuously acquired and averaged, through the use of *Get* service calls, to produce a value that is then applied, through the use of *Set* service calls, to all of the rotational module agents within the entity. The position of the rotational head of the servo motor directly assumes, within the limits of its physical rotational range, an angle proportional to the degree to which the accelerometer is offset about its *x*-axis. Likely due to its limited sensitivity, varying the ambient light incident on the LDR did not affect the angle of the servo to as great a degree as changing the orientation of the accelerometer; however, minor variations were distinctly noticeable, indicating that the voltage readings returned by the LDR were in fact influencing the average reading applied to the servo motor.

As a result of overhead encountered during the transmission and processing of the continuous stream of service calls issued by the primary module to the sensor module agents within the logical entity, reliable real-time performance was difficult to achieve. Nevertheless, the modular sensing system exhibited correct behaviour, with real-time performance limited mainly by the capabilities of the microcontroller and wireless transceiver utilized in the TIMs. Improvements in real-time performance may be attained through the utilization of a more recent variant of the ARM microcontroller as well as a transceiver capable of higher sustained transmission speeds in a newer version of the TIM hardware. The cost of such attaining such components for prototyping purposes is rapidly falling to reasonable levels, and these components will facilitate greatly reduced latencies in scenarios where service calls are continuously invoked.

### 9.3. Physical Collaboration Evaluation Setup and Procedure

The purpose of this test is to evaluate the behaviour of a modular sensing system when its constituent TIMs are physically connected. A modular actuator system will be created in which two 16 × 4 character HD44780-based liquid-crystal display (LCD) TIMs (designated Modules A and B) are physically connected in various orientations. A breadboard is also used within the experiment solely to provide an interface to a single 5 V adaptor to provide power and an RS232 port connected to a personal computer workstation for administrative purposes. As with the wirelessly interacting composite module test, the components on this breadboard otherwise have no influence on the behaviour of the composite system.

After being placed within range of each other, the LCD modules are expected to detect each other and attempt to form a composite entity. Upon connection, a composite system should be created that finds and utilizes all of the available text displays (the two LCD TIMs) in order to form a suitable logical entity that effectively functions as a larger display if the displays detect that they are connected in a suitable orientation, as per the provided template specification and template class.

To test if the described system behaviour is realized, the LCD TIMs are connected together in four configurations such that the LCD displays on the modules are aligned in suitable horizontal and vertical orientations. In the horizontal configurations, the two 16 × 4 character LCD displays should form and behave as a logical 32 × 4 LCD display, thus possessing double the width. In the vertical configurations, the two 16 × 4 character LCD displays should form and behave as a logical 16 × 8 LCD display, thus possessing double the height. Once formation of a logical module entity is indicated through confirmation received on the administrative interface, alphanumeric text strings are then transmitted through the administrative interface to the logical module. The text output on the LCD displays is then examined and analyzed in order to determine the correlation between the orientations of the LCD display modules and the text outputs observed.

### 9.4. Physical Collaboration Results and Analysis

**Figure 20.**
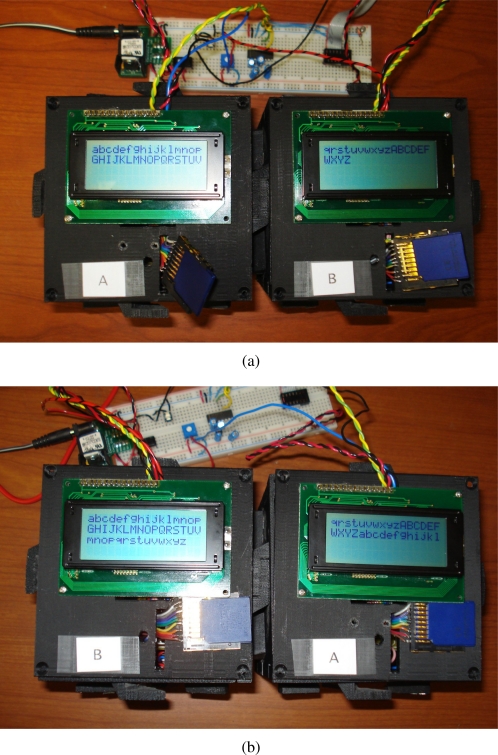
32 × 4 character composite LCD TIM configurations. **(a)** Face 2 of Module A connected to Face 4 of Module B. **(b)** Face 2 of Module B connected to Face 4 of Module A.

**Figure 21.**
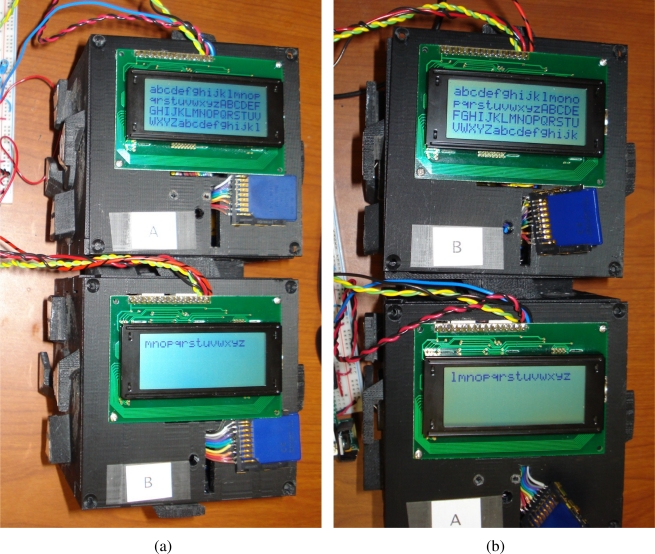
16 × 8 character composite LCD TIM configurations. **(a)** Face 5 of Module A connected to Face 3 of Module B. **(b)** Face 5 of Module B connected to Face 3 of Module A.

Figures 20 and 21 depict the behaviour of the composite sensing system formed in the second evaluation setup. As long as the LCD displays associated with the member TIMs are aligned horizontally or vertically alongside each other in a common plane, the alphanumeric string is always displayed in a consistent left-to-right, top-to-bottom fashion across TIMs. This behaviour is realized even if the relative positions of Module A and Module B are swapped. Since the member TIMs are physically connected, they possess a common pose base, and the template class executing on primary module B is therefore able to query and analyze their *pose vectors* (see Figure 16), derived from their respective pose matrices, in order to determine their relative orientations and thus the overall geometry of the logical entity. With knowledge of the overall geometry, the original alphanumeric string is internally split (if necessary) into segments by the primary module, each of which is recursively transmitted using *Set* service calls to the appropriate member LCD TIMs in order to achieve the correct behaviour.

Due to limitations in the amount of memory available within the TIMs, which restrict the complexity of the template classes utilized by logical module agents as well as the size of the structures used to maintain the state of the logical module agents themselves, the template class utilized in this test is unable to scale beyond two LCD modules. Nevertheless, the logical module entity exhibited the ability to assume a new behaviour based on the relative orientations between its physically connected member modules, and by extension, the overall geometry of the composite entity. Through the utilization of TIMs possessing greater amounts of memory (which is quickly becoming less cost prohibitive as semiconductor fabrication techniques improve), scaling to three LCD modules and beyond would not be difficult to achieve.

### 9.5. Collaboration Performance Analysis

In this subsection, measurements obtained during the evaluation of various performance criteria in the wireless and physical collaboration tests are graphically presented. For each criterion, the maximum, minimum, and mean performance readings are shown, as well as the standard deviation from the mean. At least one hundred and up to three thousand event occurrences are timed, logged, and analyzed during the collaboration test runs. These readings are then analyzed in order to characterize the performance of the architecture in each case.

#### 9.5.1. Channel Reservation Latencies

**Figure 22.**
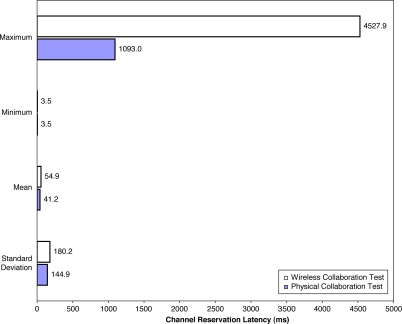
MAC protocol channel reservation latencies.

Figure 22 depicts the MAC protocol channel reservation latencies encountered at the communication layer during the collaboration tests. This latency is defined as the time elapsed between the transmission of an RTS packet and reception a CTS packet in response. The results show that the time required to reserve a channel is on the order of tens of milliseconds. In comparison, the channel reservation latencies encountered in typical 802.11/WiFi networks are typically around 20 ms when few nodes are contending for access to the wireless channel [32]. The relatively large channel reservation period typically encountered during the operation the software architecture can likely be attributed to the greatly reduced interrupt response due to frequent usage of *critical sections* during timing-critical operations. Critical sections are facilitated through the disabling of interrupts, during which reception of medium allocation packets by the wireless transceiver may go undetected for a substantial period of time. The impact of the large channel reservation overhead is somewhat mitigated by the fact that it occurs only once per message transmission, is independent of the message length, and only becomes a major issue in real-time scenarios in which data is streamed between TIMs.

#### 9.5.2. Message Transmission Speeds

**Figure 23.**
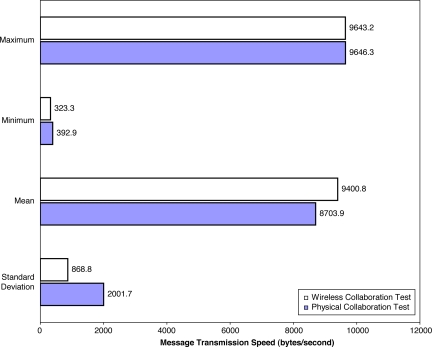
PAR protocol message transmission speeds.

Figure 23 depicts the speeds at which messages are wirelessly transmitted using the PAR protocol at the communication layer during the collaboration tests. The results clearly show a maximum transmission speed of about 9.42 kBps (kilobytes per second), with mean transmission speeds approaching this maximum. The generally larger messages transmitted during the physical collaboration test (due to the relatively lengthy alphanumeric strings) resulted in increased packet retransmissions, and thus a lower mean message transmission speed as well as a noticeably larger transmission speed variance during that test. The 9.42 kBps maximum transmission speed is much lower than the specified theoretical maximum of 2 Mbps (megabits per second), or 256 kBps, attainable by the nRF24L01 transceiver. This performance discrepancy may be attributed to the speed of the SPI bus linking the microcontroller and the transceiver, acknowledgement packet transmission overhead, and packet encryption/decryption overhead. The maximum message transmission speed of 9.42 kBps is, however, very well suited to a wide variety of sensor-actuator systems and applications.

#### 9.5.3. Service Call Round-Trip Latencies

**Figure 24.**
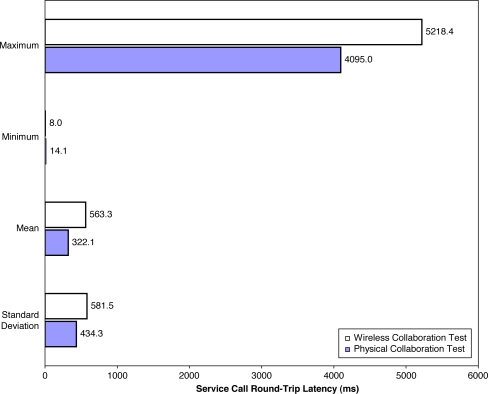
Service call round-trip latencies.

Figure 24 depicts the latencies encountered during the invocation of synchronous *Call By* service functions in the collaboration tests. This latency is defined as the time elapsed, or *round-trip interval*, between the transmission of a service call message and the reception of the associated return message. Service calls that are asynchronous or *Call At* calls are not considered since such service calls are typically subject to lengthy and/or widely varying latencies, and will be utilized less often in practice than synchronous *Call By* service calls.

The results show that the return message reception latencies for synchronous *Call By* service function invocations are typically around 300 to 500 ms on average. In comparison, the round-trip latencies encountered during the operation of the standard *Remote Procedure Call* (RPC) protocol usually employed on high-speed wired and wireless networks are typically below 100 ms for small messages [33]. The relatively large round-trip latency at the middleware layer can noticeably limit the ability of the TIMs (in their current form) to be applied to real-time streaming applications. In addition to the previously outlined factors that impact channel reservation latencies and message transmission speeds (which in turn impact the speed of service function invocations), a major factor impacting message round-trip latencies is the substantial overhead encountered in the interpretation of Java bytecodes during service calls.

#### 9.5.4. Bytecode Execution Speeds

**Figure 25.**
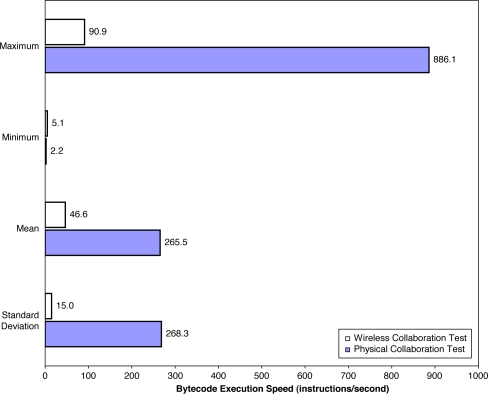
Virtual machine bytecode execution speeds.

Figure 25 depicts the speeds at which the Java virtual machine bytecode instructions comprising logical module template classes are interpreted and executed at the virtual machine layer. The mean and maximum bytecode execution speeds (47 and 91 instructions/second respectively) attained during the wireless collaboration test are noticeably lower than the mean speed of about 266 instructions/second attained during the physical collaboration test. This discrepancy may be attributed to the much higher incidence of synchronous service calls during the wireless collaboration test, which result in the frequent suspension of template class execution.

The attained bytecode interpretation speeds during the collaboration tests are considerably slower (on the order of thousands of times) than native machine code execution. The microcontroller clock speeds and limited memory available in the TIM hardware are not conducive to popular acceleration techniques such as *just-in-time recompilation* (JIT) [34]. Newer versions of the ARM processor core overcome these performance limitations by supporting the execution of Java bytecodes directly in hardware, through the use of the *Jazelle* [34] architecture extension.

#### 9.5.5. Startup Memory Utilization

**Figure 26.**
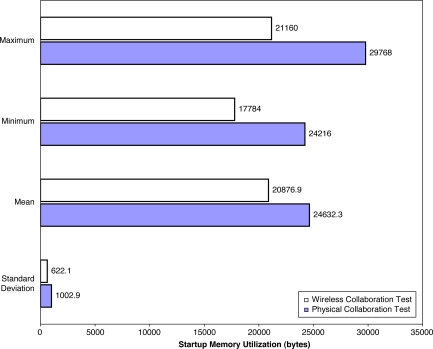
Startup memory utilization after logical module creation.

Figure 26 depicts the amount of random access memory (RAM) utilized on the TIMs (out of the 31 kilobytes integrated into the LPC2148 microcontroller) by the software architecture upon formation of the logical modules within each collaboration test. The results clearly show that about 20 to 24 KB (kilobytes) on average, or about 65% to 77% of the available memory, is typically utilized upon logical module formation. The consistently larger memory utilization by the physical collaboration test may be attributed to the larger and more complex template class utilized in the test. Due to the fact that complex template classes will often require on the order of 4 KB of RAM or more, scalability of logical module combinations is limited. Nevertheless, the software architecture is designed to scale with increasing amounts of processing power and available memory, which is quickly becoming less cost prohibitive with improvements in semiconductor fabrication techniques.

## 10. Conclusions

### 10.1. Concluding Remarks

This paper presented a novel software architecture and knowledge representation scheme that facilitates the flexible, scalable, and reliable combination of heterogeneous modular sensor and actuator components.

The main feature of this architecture is the *composition* system that allows a sensor to be combined with an actuator, thereby augmenting the sensor with motion capability and enabling the now *active* sensor to adapt to changing process requirements. Each modular component, known as a *TIM*, provides a core sensing or actuation functionality and also possesses embedded knowledge of its capabilities, in the form of a *TEDS* specification, which may be communicated to other modules in its environment. This facilitates collaboration among a group of sensor and actuator modules, and enables all or a subset of the group to dynamically exhibit completely new behaviour. This dynamic reprogramming intelligence is provided in the form of hardware-independent, Java-based *template classes*. A lightweight, customized Java virtual machine specific to this software architecture was implemented in order to support the dynamic reprogramming of TIMs containing highly resource-constrained embedded hardware. Such hardware is incapable of running the traditional, much more resource intensive Java virtual machines.

The proposed software architecture was implemented and evaluated using a prototype transducer module implementation in order to test its viability, and this work is a first step towards a highly adaptive architecture that will prove useful in applicable domains such as flexible inspection, mobile robotics, surveillance, and even space exploration.

### 10.2. Recommendations

Although the adaptive modular sensing system software architecture exhibited correct behaviour when implemented and evaluated on actual TIM hardware, a number of areas in the design and implementation of the software architecture may be improved, and new features may be added, in order to increase its applicability.

Processing and memory limitations imposed by the current TIM hardware implementation introduced relatively large latencies into multiple layers of the software architecture, limiting its applicability to demanding scenarios where real-time performance is required. Utilizing a revision of the TIM hardware possessing a microcontroller based on more recent, high-speed versions of the ARM processor, such as those in the ARM9 and ARM11 series, would result in substantially improved architecture performance and allow the usage of highly bandwidth-intensive sensors such as embedded cameras.

Greater sustained packet and message transmission speeds would also noticeably improve the performance of the overall software architecture, particularly in scenarios requiring real-time collaboration between member modules transferring substantial amounts of data, since the round-trip latency for every service call issued would be reduced. Moving channel reservation and switching logic, as well as packet encryption and decryption, into the firmware of the transceiver would also aid in reducing these latencies. Utilizing a *Bluetooth* [35] transceiver would be well suited to achieving these goals.

Throughout the software architecture, *critical sections* are extensively utilized to facilitate the atomic operation of timing-critical operations, which has the side effect of preventing the real-time operating system (RTOS) from performing context switches between concurrently executing tasks. With frequent utilization, critical sections often result in greatly increased execution latencies. On hardware possessing greater processing capability, *mutexes* and *semaphores* should be utilized to implement task-safe access to shared data without greatly reducing execution latencies.

To keep the virtual machine within the software architecture lightweight, features of complete Java virtual machine implementations deemed non-critical, such as threads and automatic garbage collection, were not implemented. On more capable TIM hardware, these features should be provided for increased flexibility. Alternatively, more resource-intesive interpreters for well-supported and well-documented scripting languages such as *Lua* [36] and *Python* [37] may be incorporated in place of the Java-based virtual machine. Template scripts would be interpreted on the fly, and would thus be easier to implement, deploy, and debug.

The library of template TEDS specifications and processing algorithms local to each TIM within a modular sensing system is updated in an automatic, peer-to-peer fashion. However, no mechanism is provided by which only the latest version of a particular template TEDS specification and its associated template class is transferred between TIMs before execution. A versioning scheme should be incorporated into TEDS specifications to ensure that a module with the latest version of a particular specification (and its associated template class if applicable) does not have its stored specification overwritten by older versions during peer-to-peer updates.

In summary, implementing and evaluating the software architecture proposed in this thesis has enabled numerous performance limitations to be exposed that would otherwise be difficult to discover. Addressing the latencies encountered at multiple layers within the software architecture stack as well as improving the speeds of message transmission and bytecode execution will result in a greatly improved adaptive modular sensing system architecture that will prove useful in its many applicable domains.
